# Appointment

**Published:** 1862-10

**Authors:** 


APPOINTMENT.—Dr. Edward Stork, of Buffalo, has been appointed Surgeon to the N. Y. S. V., with expectation of service in the new regiment now being raised in Buffalo. We congratulate the regiment upon securing the seryices of so worthy a surgeon.

